# Semaglutide-Induced Acute Pancreatitis Leading to Death After Four Years of Use

**DOI:** 10.7759/cureus.69704

**Published:** 2024-09-19

**Authors:** Chebly Dagher, Mohamed Jailani, Maria Akiki, Talha Siddique, Zidan Saleh, Evan Nadler

**Affiliations:** 1 Internal Medicine, University of Connecticut, Farmington, USA; 2 Pulmonary and Critical Care, St. Francis Hospital, Hartford, USA

**Keywords:** acute respiratory distress syndrome [ards], anuric acute kidney injury, distributive shock, glp-1 ra, medication-induced pancreatitis, severe pancreatitis

## Abstract

Glucagon-like peptide-1 receptor agonists (GLP-1RAs) are essential in managing type 2 diabetes mellitus, promoting glucose regulation, weight reduction, and cardiovascular protection. Here, we report a unique case of semaglutide-induced pancreatitis complicated by distributive shock and death after four years of use.

A 74-year-old male with type 2 diabetes, atrial fibrillation, coronary artery disease, and obesity (BMI 31.7) presented with severe epigastric pain and was diagnosed with severe pancreatitis. He reported no recent alcohol, drug use, or abdominal trauma. His workup showed normal triglyceride and calcium levels, and abdominal ultrasound was negative for gallstones or choledocholithiasis. He had been on semaglutide for four years, with a dose increase from 0.25 to 0.5 mg weekly about four weeks prior to admission, which was associated with worsening side effects such as severe nausea and constipation. He was admitted to the intensive care unit for severe pancreatitis, which was complicated by distributive shock requiring vasopressors, renal failure requiring continuous renal replacement therapy, Acute respiratory distress syndrome (ARDS) requiring intubation, and subsequently, cardiac arrest. In this case, the patient developed severe acute pancreatitis leading to death after four years of GLP-1RA use, with a dose increase occurring four weeks prior to admission. The absence of typical risk factors suggests a potential link between long-term use and dose changes of GLP-1RAs and severe pancreatitis.

## Introduction

Glucagon-like peptide 1 (GLP-1) is a natural hormone released from the lower digestive system that boosts insulin levels when glucose is taken orally rather than intravenously in healthy individuals. Additionally, GLP-1 decreases glucagon levels, delays gastric emptying, and reduces appetite [1]. Accordingly, GLP-1 receptor agonists (GLP-1RA) have emerged as crucial medications in managing type 2 diabetes mellitus by enhancing glucose regulation, promoting weight reduction, and offering protection against cardiovascular diseases [2].

Exenatide was the first GLP-1RA to receive FDA approval in 2005 for the management of type 2 diabetes mellitus, and since then, multiple similar medications have been developed. Currently, various GLP-1RA are employed in the treatment of type 2 diabetes [3]. Ozempic, also known by its generic name semaglutide, received FDA approval for the management of type 2 diabetes mellitus in 2017 [4]. Recently, semaglutide has also been approved for use in patients with obesity and is sold under the name Wegovy^TM^ [5].

GLP-1 receptor agonists have been linked to the development of acute pancreatitis and are suggested to potentially cause pancreatic cancer. However, longer-term cardiovascular outcome trials (CVOTs) and phase 3 studies, including those focused on semaglutide, have not shown significant differences in the incidence of pancreatic adverse events compared to placebo, though the follow-up duration may not be sufficient to fully assess long-term risks [6]. Other studies have shown conflicting results, indicating an increased risk of pancreatitis [7].

Pancreatitis associated with semaglutide may be under-reported because diabetic patients who qualify for GLP-1RA therapy often have other risk factors for acute pancreatitis, such as obesity, a longer duration of diabetes, and the use of other medications that independently increase pancreatitis risk. Most case reports indicate acute pancreatitis as a side effect shortly after semaglutide exposure. Here, we report a case of semaglutide-induced pancreatitis that occurred after four years of use.

## Case presentation

The patient is a 74-year-old male with type 2 diabetes mellitus, hypertension, atrial fibrillation, coronary artery disease, obstructive sleep apnea, and obesity, with a BMI of 31.7. His long-standing outpatient medications included amlodipine, atorvastatin, metoprolol, eplerenone, apixaban, allopurinol, insulin degludec, and semaglutide.

He presented to the emergency department at our institution with complaints of sudden-onset abdominal pain that developed approximately 12 hours earlier. The patient described severe epigastric pain radiating to his back, which had started while eating dinner. This was followed by continuous non-bloody, non-bilious vomiting. He had tried to take antacids to relieve the pain, but they did not help. He denied fevers, chills, and consumption of stale food. Additionally, he denied shortness of breath, chest pain, diarrhea, or changes in urinary frequency. The patient had been abstinent from alcohol for several months, did not use tobacco, and had never used recreational drugs. He denied any abdominal injury. His only prior surgery was a coronary artery bypass graft approximately six years ago.

Regarding his diabetes, he was diagnosed 20 years ago and was managed with an insulin regimen and metformin until about four years ago. At that time, his hemoglobin A1C was above goal (7.8%), and given his concurrent obesity (body mass index 38), semaglutide was started at a dose of 0.25 mg weekly. He was maintained on that dose for a while before it was increased to 0.5 mg weekly, which resulted in a good response, with a steady decline in his weight and improvement in hemoglobin A1C. Approximately four weeks prior to admission, he complained of severe constipation, nausea, and vomiting, and accordingly, the semaglutide dose was reduced to 0.25 mg weekly.

On presentation, the patient was vitally stable and on room air. On examination, he had diffuse abdominal tenderness, significantly worse in the epigastric area. Laboratory investigations showed leukocytosis of 18,5 x 10^9^/L and elevated C-reactive protein (CRP) to 15.2 mg/dl. He had a blood urea nitrogen (BUN) of 45 mg/dL and creatinine of 2.9 mg/dL, which was an increase from 1.2 mg/dL a few weeks earlier. Liver enzymes were as follows: aspartate aminotransferase (AST) at 90 IU/L and alanine aminotransferase (ALT) at 29 IU/L. Albumin was 3.4 g/dl. Lipase was significantly elevated at 3316 U/L and amylase at 507 U/L. Triglycerides were normal at 84 mg/dL. All laboratory values are summarized in Table 1.

**Table 1:** Summary of the patient's laboratory results mmol/L: millimoles per liter; mg/dL: milligrams per deciliter; U/L: units per liter; %: percent; /L: per Liter; g/dL: grams per deciliter.

	Values	Reference range
White blood cells	18.5	4.5-11 10*9/L
Creatinine	2.9	0.7 - 1.3 mg/dL
Blood Urea Nitrogen (BUN)	45	9 - 20 mg/dL
Sodium	131	135 - 145 mmol/L
Carbon Dioxide	17	24 - 32 mmol/L
Calcium	8	8.4 - 10.2 mg/dL
Potassium	5.9	3.5 - 5.1 mmol/L
Aspartate aminotransferase (AST)	90	10-40 U/L
Alanine aminotransferase (ALT)	29	7-52 U/L
Lipase	3316	11-82 U/L
Triglyceride	84	<150 mg/dL
Hemoglobin A1c	6.5	4-6%
Albumin	3.4	3.5 - 5.0 g/dL
Amylase	507	29 - 103 U/L

**Figure 1:**
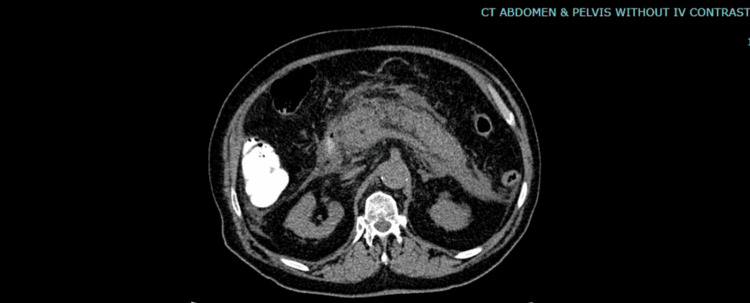
Computed tomography image of the abdomen and pelvis (without contrast) The image shows a swollen pancreas with no low attenuation areas with hazy and streaky induration surrounding it.

Computed tomography of the abdomen and pelvis revealed free intraperitoneal fluid along the liver edge and in both paracolic gutters with hazy, streaky induration surrounding the pancreas. The pancreas appeared swollen with no low attenuation areas. A few nodes were seen in the porta hepatis. There were no gallstones or choledocholithiasis. Findings were most consistent with pancreatitis with adjacent phlegmon and inflammatory changes in both paracolic gutters and fluid over the liver (Figure 1).

Accordingly, his Acute Physiology and Chronic Health Evaluation (APACHE) II score was 25% for estimated nonoperative mortality. To prognosticate his outcomes, the Bedside Index for Severity in Acute Pancreatitis (BISAP) score was 2, which indicated an increasing risk of mortality.

The patient was admitted to the Intensive Care Unit for management of semaglutide-induced acute pancreatitis. He was started on intravenous fluids, pain management, and empiric antibiotics. In the first 24 hours in the ICU, his hospital course was complicated by progressive hypotension that required the start of vasopressors, initially with norepinephrine. This led to worsening renal function, eventually progressing to anuric kidney injury and worsening hyperkalemia. He was initiated on continuous renal replacement therapy.

After 48 hours of admission, he developed hypoxia with increasing oxygen requirements until the third day of presentation, when he was intubated. Shortly after intubation, the patient had a cardiac arrest and, post-arrest, required maximum doses of vasopressors, including norepinephrine, epinephrine, phenylephrine, and vasopressin. Given his poor overall prognosis and after intensive discussions with family members, the patient was transitioned to a comfort care management plan. 

## Discussion

Subcutaneous semaglutide, a glucagon-like peptide-1 receptor agonist, is gaining popularity for glycemic control in type 2 diabetes mellitus patients due to its lower risk of hypoglycemia, weight loss benefits, and convenient weekly injection [8]. Semaglutide, like any medication, has side effects. These adverse events have been addressed through extensive phase 3 registration trials, including cardiovascular outcome trials for both its subcutaneous (SUSTAIN) and oral (PIONEER) formulations [9], including gastrointestinal symptoms, acute kidney injury, gallbladder issues, thyroid cancer, and pancreatic complications like pancreatitis and pancreatic cancer [10].

A few years after GLP-1RA was introduced, these agents were associated with incidents of acute pancreatitis [11]. It is hypothesized that the direct activation of glucagon-like peptide 1 receptors on pancreatic exocrine duct cells and pancreatic islet beta cells stimulates the growth and proliferation of these cells, which results in increased pancreatic mass and blockage of the exocrine ducts leading to acute pancreatitis [12]. Sodhi et al. conducted a study on 16 million individuals, which showed that the incidence of pancreatitis was higher in patients using semaglutide and liraglutide compared to those using bupropion-naltrexone for weight loss [7]. Also, several case reports described cases of acute pancreatitis secondary to semaglutide use. Patel et al. described a case of a 61-year-old diabetic patient on semaglutide for two months who presented to the emergency department with severe epigastric pain and was found to have acute pancreatitis [13]. Araiza et al. described the case of a 51-year-old female with a medical history of obesity, who had started weight-loss treatment with semaglutide 12 weeks prior. She presented with one day of severe epigastric pain radiating to her back, associated with several episodes of emesis, and was found to have acute necrotizing pancreatitis [14].

However, other studies had contradictory findings. For example, in the SUSTAIN 6 trial, acute pancreatitis was reported in nine patients treated with semaglutide and in 12 patients treated with placebo [15]. In PIONEER 6, acute pancreatitis was identified in one patient treated with semaglutide, compared to three patients treated with placebo [16]. 

Our patient had a medical history of Class I obesity and type 2 diabetes mellitus, both known risk factors for acute pancreatitis. The patient had been on semaglutide 0.25 mg weekly for four years, but a few weeks before presentation, the dose was increased to 0.5 mg weekly, leading to severe constipation, nausea, and vomiting. Consequently, the dose was reduced back to 0.25 mg. The patient was unable to tolerate higher doses of semaglutide and subsequently presented with acute pancreatitis. Notably, the patient did not report typical risk factors for acute pancreatitis such as recent abdominal trauma, alcohol intake, corticosteroid use, or infections. The patient had not started taking new medications or used any supplements or herbal substances. Additionally, laboratory tests did not show hypercalcemia or hypertriglyceridemia, and imaging studies did not reveal stones in the biliary tract. Given the patient's complaint of worsened common side effects like constipation, nausea, and vomiting following the increase in GLP-1RA dose, which was subsequently reduced before the onset of pancreatitis, there is suspicion that GLP-1RA induced pancreatitis may be involved. This raises the question of whether patients experiencing worsening common side effects of GLP-1 agonists are at higher risk for pancreatitis.

## Conclusions

Most case reports indicate acute pancreatitis as a side effect shortly after Semaglutide exposure. To our knowledge, no cases of acute pancreatitis have been reported after four years of semaglutide use or after a recent increase in semaglutide dose, making our case unique. Further studies are needed to investigate the possibility of late-onset pancreatitis as a side effect of semaglutide.
